# Psychological Flexibility Moderates the Association Between Multidimensional Stress and Psychological Distress in Medical Postgraduates: A Multi-Center Cross-Sectional Study

**DOI:** 10.3390/bs16030374

**Published:** 2026-03-06

**Authors:** Yuan Lai, Yu Gu, Yanqi Chen, Zhengjuan Hu, Wen Zheng

**Affiliations:** 1 Graduate School, Capital Medical University, Beijing 100069, China; laiyuan-xinli@ccmu.edu.cn; 2 School of Marxism, Capital Medical University, Beijing 100069, China; 3 School of Public Health, Capital Medical University, Beijing 100069, China; 4 Department of Medical Psychology, School of Medical Humanities, Capital Medical University, Beijing 100069, China; chenyanqi1230@foxmail.com

**Keywords:** mental health, stress, psychological flexibility, medical postgraduate students, cross-sectional research

## Abstract

The mental health issues of medical postgraduate students are increasingly prominent, and it is urgently necessary to explore the stressors and protective factors. This study adopted an integrative approach to examine the moderating role of psychological flexibility in the relationship between multiple types of life stress and mental health. A total of 5819 medical postgraduate students from a medical university and its affiliated hospitals in Beijing were surveyed in a multi-center cross-sectional study. Measures included psychological worry, supervisory relationship, work–life balance, school support, psychological flexibility, and psychological health. The results showed that all four types of stressors were significantly associated with mental health. Psychological worry was positively associated with psychological distress, while the other three variables were negatively associated with it. Psychological flexibility was negatively associated with psychological distress and the relationship between each stressor and psychological health was weaker with higher levels of psychological flexibility. These findings highlight the heterogeneity in the pathways by which different stressors affect psychological health, underscore the critical role of psychological flexibility in coping with internally generated cognitive stress, and provide theoretical and practical implications for psychological interventions among medical postgraduates.

## 1. Introduction

In recent years, the mental health of medical postgraduates has become an increasingly prominent issue and a pressing challenge in medical education. As individuals who simultaneously assume the dual roles of “doctor” and “student,” medical postgraduates experience substantial psychological stress stemming from the multiple demands of clinical training, scientific research, and career development (Al Saadi et al., 2017; Dyrbye & Shanafelt, 2011). A growing body of empirical evidence indicates that the prevalence of anxiety and depression among medical postgraduates is markedly higher than that of the general postgraduate population. The global prevalence of depressive symptoms among medical students was 27.2%, and the overall prevalence of suicidal ideation was 11.1% (Rotenstein et al., 2016). A more recent meta-analysis in 2019 among medical students in China revealed a mean of 32.74% for depression prevalence, and a mean of 27.22% for anxiety prevalence. (Mao et al., 2019).

Despite these alarming findings, few studies have examined, from an integrative perspective, whether the various stressors faced by medical postgraduates influence mental health. Furthermore, potential individual-level psychological resources that may buffer the adverse effects of stress have not been adequately explored. Against this backdrop, psychological flexibility has recently attracted increasing scholarly attention as a positive psychological trait (Guo et al., 2022). Existing studies suggest that psychological flexibility may mitigate the sustained impact of negative experiences by enhancing coping efficacy and emotion regulation capacity (Guo et al., 2022). Therefore, investigating the moderating role of psychological flexibility in the relationship between multidimensional stress and mental health among medical postgraduates is of both theoretical and practical significance, thus deepen our understanding of adaptive factors in this population and provide a theoretical foundation for precision-based psychological interventions in medical education.

### 1.1. Graduate Stress and Mental Health: The Unique Situation of Medical Postgraduates

The postgraduate stage is widely regarded as a critical transitional period for academic development and professional transformation, during which individuals experience a substantial increase in psychological stress (Dyrbye & Shanafelt, 2011). Medical postgraduates, in particular, face a more complex and fragile mental health situation than those in other disciplines, as they must simultaneously manage multiple demanding tasks, including clinical rotations, scientific research, and academic requirements (Cohen & Patten, 2005). Within the current integrated medical education–clinical training system, these students occupy dual roles as both learners and frontline medical practitioners. Consequently, they experience greater role conflict and time pressure compared to their non-medical peers (Kelly et al., 2020).

The sources of psychological stress among medical postgraduates can be broadly categorized into several domains. First, academic-related stress is prevalent, encompassing worries about research progress, academic performance, and graduation requirements (Gaston-Hawkins et al., 2020; Guo et al., 2022). Second, the supervisor–student relationship has been identified as a salient psychosocial risk factor (Althubaiti & Althubaiti, 2022). Previous studies demonstrated that academic and emotional support from supervisor significantly alleviates graduate stress, and perceived stress from supervisors not only directly contributes to research burnout but also indirectly exacerbates psychological distress by weakening professional identity (Wu et al., 2024). Third, medical postgraduates often struggle to maintain work–life balance due to frequent transitions between intensive clinical rotations and research activities (Kelly et al., 2020). Issues such as unpaid overtime and ambiguous clinical roles are common in medical education, leaving students exhausted and with limited opportunities for rest and self-recovery (Gaston-Hawkins et al., 2020). Finally, institutional support deficiencies (e.g., inadequate psychological counseling services, ambiguous financial aid systems, and unequal access to academic resources, etc.) serve as hidden stressors that diminish psychological resilience and a sense of belonging among medical postgraduates (Ye et al., 2023).

Taken together, the psychological challenges faced by medical postgraduates are not only more severe in degree but also more complex and persistent in nature. The present study identifies four common categories of stressors in the daily lives of medical postgraduates as core predictive variables: academic worry, supervisor–student relationship, work–life balance, and institutional support. Furthermore, it examines the pathways through which these stressors influence mental health and investigates the moderating role of psychological flexibility in these associations. By doing so, this study aims to provide a structured theoretical framework to inform the design of targeted psychological interventions for medical postgraduates.

### 1.2. Psychological Flexibility and Mental Health: Direct and Indirect Pathways

Psychological flexibility has emerged as a central construct in recent clinical and health psychology research. It originates from the core theoretical framework of the “third wave” of cognitive-behavioral therapies, namely Acceptance and Commitment Therapy (ACT; S. C. Hayes, 2004; Stockton et al., 2019). ACT emphasizes that when facing negative emotions and life stress, individuals can achieve psychological adaptation and well-being by accepting their present experiences, disengaging from unhelpful cognitive fusion, and acting in accordance with personally held values (Stockton et al., 2019). According to ACT theory, psychological flexibility comprises six interrelated functional processes: acceptance, cognitive diffusion, contact with the present moment, self-as-context, values clarification, and committed action (S. C. Hayes, 2004). These processes work synergistically to enable individuals to maintain behavioral consistency and adaptive coping in the face of emotional, cognitive, and environmental stressors.

A substantial body of empirical evidence supports the crucial role of psychological flexibility in promoting mental health (Lucas & Moore, 2020; Stockton et al., 2019). Psychological flexibility negatively predicted negative emotions, and positively predicted subjective well-being among college students (Grégoire et al., 2020; Marshall & Brockman, 2016) and other populations (Grégoire et al., 2018). Beyond its direct effects, psychological flexibility has been widely demonstrated to exert an important moderating role in the relationship between stress and mental health. Individuals with higher levels of psychological flexibility are better able to maintain emotional stability and overall well-being under conditions of external stress. For instance, psychological flexibility significantly attenuated the negative impact of perceived stress on medical staff working in intensive care settings (Holmberg et al., 2020). Moreover, psychological flexibility has been identified as a potential protective variable across various stress–health pathways, including psychedelics use, depression, and anxiety (Davis et al., 2020; Grégoire et al., 2018).

However, existing empirical studies have primarily focused on clinical populations, such as individuals with chronic pain, anxiety, depression, or addiction (Stockton et al., 2019), with relatively limited research on non-clinical populations experiencing sustained high levels of stress, such as medical postgraduates. Moreover, it remains unclear the effect of psychological flexibility under the intersection of multiple academic and developmental stressors in relation to mental health outcomes. At the same time, research on graduate students has consistently shown that multidimensional life stress, such as worries about research progress, tense supervisor–student relationships, work–life imbalance, and inadequate institutional support, are major predictors of their mental health status. A key question for current research, therefore, is how to identify and activate the psychological resources that enable individuals to withstand these stressors. Given the potential heterogeneity among stressors, the potential buffering effect of psychological flexibility may not be uniform across all stress–health pathways. These questions warrant systematic empirical investigation.

### 1.3. The Present Study

The present study takes four core categories of life stress commonly experienced by medical postgraduates (i.e., academic worry, supervisor–student relationship, work–life balance, and institutional support) as predictors, and examines their respective effects on mental health. The moderating effects of psychological flexibility on the associations between these stressors and mental health were further examined through statistical analysis. A cross-sectional survey design was adopted. By addressing these questions, this study aims to provide a theoretical foundation and practical direction for developing psychological support systems tailored to the needs of medical postgraduates.

Based on the theoretical framework and empirical evidence discussed above, the present study proposes the following hypotheses:

**Hypothesis** **1.** *Academic worry, supervisor–student relationship, work–life balance, and institutional support are each significantly and positively associated with psychological distress among medical postgraduates.*

**Hypothesis** **2.** *Psychological flexibility significantly and negatively predicts the level of psychological distress among medical postgraduates.*

**Hypothesis** **3.** *Psychological flexibility moderates the effects of life stress (academic worry, supervisor–student relationship, work–life balance, and institutional support, respectively) on psychological distress. Specifically, the negative impact of stress on mental health is expected to be weaker among individuals with higher levels of psychological flexibility.*

## 2. Materials and Methods

### 2.1. Participants

A cross-sectional survey was conducted in September 2024 using a cluster sampling method. Participants were recruited from a medical university in Beijing and its 24 affiliated hospitals, including both master’s and doctoral students. A total of 5819 questionnaires were collected. After excluding incomplete responses and those failing the validity checks (i.e., who failed the attention check items or who reported repeatedly consistent answers), 3753 completed total questionnaires were retained for analysis, yielding an effective response rate of 64.6%.

This study was reviewed and approved by the Ethics Committee of Capital Medical University (Approval No. 2024SY243). All participants provided informed consent prior to completing the survey.

Among the valid respondents, 1178 were male (31.4%) and 2575 were female (68.6%). Participants ranged in age from 21 to 42 years (*M* = 27, *SD* = 2). The sample comprised 1577 doctoral students (42.0%) and 2176 master’s students (58.0%). In terms of degree type, 1594 (42.5%) were academic postgraduates and 2158 (57.5%) were professional track postgraduates.

### 2.2. Measures

#### 2.2.1. Symptom Checklist-90 (SCL-90)

The Symptom Checklist-90 (SCL-90; Derogatis & Unger, 2010) was used to assess psychological distress, including symptoms of depression, anxiety, stress, and somatization. The instrument consisted of 90 items rated on a 5-point Likert scale ranging from 1 (not at all) to 5 (extremely). Higher total scores indicate greater levels of psychological distress. In the present study, the internal consistency of the SCL-90 was excellent (Cronbach’s *α* = 0.96).

#### 2.2.2. Sources of Postgraduate Stress

The Postgraduate Stress and Support Questionnaire used in this study was adapted from the large-scale investigation of graduate student mental health conducted by *Nature* Career (Woolston, 2022). The instrument was open-access, consisted of 24 items covering four dimensions: (a) academic worry (e.g., “*I have fear of delayed graduation*”, (b) supervisor–student relationship (e.g., “*I can feel my supervisor’s support*”), (c) work–life balance (e.g., “*I have work autonomy*”), and (d) institutional support (e.g., “*My university provides individual psychological counseling*”). These dimensions assess both perceived stress and perceived support in postgraduate study and life (full items see Appendix A). English to Chinese translation and cultural adaptation was conducted by the author team and student representatives prior than this study to improve accuracy and community engagement.

All items were rated on a 5-point Likert scale ranging from 0 (strongly disagree) to 4 (strongly agree). The total score for each subscale represents the level of perceived stress or support in that domain. For example, higher scores on the academic worry subscale indicate greater concern about one’s academic and research progress. In the present study, the internal consistency coefficients (Cronbach’s *α*) for the four dimensions were as follows: academic worry = 0.88, supervisor–student relationship = 0.88, work–life balance = 0.89, and institutional support = 0.91.

#### 2.2.3. Psychological Flexibility

Psychological flexibility was assessed using the Comprehensive Assessment of Acceptance and Commitment Therapy Processes (CompACT; Trindade et al., 2021). The CompACT comprising 15 items across three dimensions: (a) openness to experience (acceptance and cognitive diffusion), (b) behavioral awareness (mindfulness and self-as-context), and (c) valued action (values and committed action). Items are rated on a 7-point Likert scale ranging from 0 (never true) to 6 (always true), with higher total scores indicating greater psychological flexibility. In the current study, the internal consistency coefficient (Cronbach’s *α*) for the scale was acceptable at 0.75.

### 2.3. Procedure

The survey was conducted from 5 September to 16 September 2024, via the online psychological assessment platform of a medical university in Beijing. The Graduate School of the university distributed the survey announcement, and the affiliated hospitals coordinated the data collection within the designated period.

Prior to participation, all respondents were informed about the study’s purpose and procedures, as well as their rights as participants. After logging into the platform, participants completed the SCL-90, the Postgraduate Stress and Support Questionnaire, and the CompACT. Upon completion, each participant received automated feedback summarizing their test results.

All data were anonymized prior to analysis, with personal identifiers such as names and student numbers removed to ensure confidentiality and data security.

### 2.4. Data Analysis

All data processing and statistical analyses were conducted using R (version 4.5.1). The packages *psych*, *lmtest*, and *interactions* were employed for correlation analysis, linear regression, and moderation testing, respectively. Simple slope analysis was further performed to interpret significant interaction effects. Data visualization was completed using the *ggplot2* package. Continuous variables conforming to a normal distribution were expressed as mean ± standard deviation (*M* ± *SD*).

#### 2.4.1. Factor Analysis

Prior to analysis, the suitability of the questionnaire data for exploratory factor analysis (EFA) was examined. The Kaiser–Meyer–Olkin (KMO) measure of sampling adequacy was 0.94, and Bartlett’s test of sphericity was significant (*χ*^2^ = 59,276.40, *df* = 276, *p* < 0.001), indicating that the data were appropriate for factor analysis. Using the maximum likelihood extraction method, four factors with eigenvalues greater than 1 were identified, collectively explaining 65.7% of the total variance.

Items were screened based on the following criteria: (1) factor loading less than 0.40; (2) double loadings, both greater than 0.40; and (3) fewer than three items per factor. No items met these deletion criteria, resulting in a final scale of 23 items. The four extracted factors were labeled as follows: Factor 1: Academic Worry (8 items); factor 2: Supervisor–student Relationship (6 items); factor 3: Work–Life Balance (5 items); factor 4: Institutional Support (4 items).

A confirmatory factor analysis (CFA) was subsequently conducted to validate the questionnaire structure. The four-factor model demonstrated acceptable goodness of fit: *χ*^2^/*df* = 1.60, RMSEA = 0.01, CFI = 0.99, AIC = 618.04, BCC = 621.38.

#### 2.4.2. Moderation Analysis

To examine whether psychological flexibility moderates the association between life stress and psychological health, four separate moderation models were constructed. In each model, one of the four dimensions of the Postgraduate Stress and Support Questionnaire (academic worry, supervisor–student relationship, work–life balance, and institutional support) served as the independent variable, the SCL-90 total score as the dependent variable, and psychological flexibility as the moderator. Age, gender, degree type (i.e., academic or professional), and degree stage (i.e., master or doctoral) were controlled as covariates.

All continuous variables were standardized prior to analysis. Confidence intervals were estimated using bias-corrected bootstrapping with 5000 resamples. A significant interaction term indicated that psychological flexibility exerted a moderating effect on the respective stress–health pathway. Please see Supplementary Material for anxiety and depression as outcomes.

## 3. Results

### 3.1. Relationships Among Sources of Postgraduate Stress, Psychological Health, and Psychological Flexibility

Pearson correlation analyses were conducted to examine the associations among psychological flexibility, academic worry, supervisor–student relationship, work–life balance, institutional supports, and psychological distress (as measured by the SCL-90 total mean score). The results are presented in Table 1.

Psychological flexibility was significantly and negatively correlated with the psychological distress (*r* = −0.32, *p* < 0.001), indicating that students with higher psychological flexibility reported fewer overall psychological symptoms. Among the four dimensions of the Postgraduate Stress and Support Questionnaire, significant correlations were also observed with psychological distress. Specifically, academic worry was positively associated with the psychological distress (*r* = 0.47, *p* < 0.001), whereas supervisor–student relationship (*r* = −0.30, *p* < 0.001), work–life balance (*r* = −0.30, *p* < 0.001), and institutional supports (*r* = −0.31, *p* < 0.001) were negatively associated with psychological distress.

These findings suggest that greater academic worry is related to higher levels of psychological symptoms, whereas better supervisor–student relationships, greater work–life balance, and stronger institutional support are associated with lower levels of psychological distress.

### 3.2. The Moderating Role of Psychological Flexibility in the Relationship Between Postgraduate Life Variables and Mental Health

#### 3.2.1. Psychological Flexibility as a Moderator of the Relationship Between Academic Worry and Psychological Health

The regression model examining the moderating role of psychological flexibility between academic worry and psychological health was statistically significant, *R*^2^ = 0.28, *F*(3, 3749) = 479.68, *p* < 0.001, indicating that approximately 27.7% of the variance in the SCL-90 total score was explained by the model. Academic worry was positively associated with psychological distress (β = 0.42, *SE* = 0.02, *t* = 27.92, *p* < 0.001, η^2^ = 0.16), whereas psychological flexibility was negatively associated with psychological distress (β = −0.14, *SE* = 0.01, *t* = 3.36, *p* < 0.001, η^2^ = 0.12). Among covariates, male gender (β = 0.03, *SE* = 0.02, *t* = 27.92, *p* < 0.001) and master degree stage (β = −0.04, *SE* = 0.01, *t* = −3.65, *p* < 0.001) were significantly associated with psychological distress.

More importantly, the interaction between academic worry and psychological flexibility was significant (β = −0.17, *SE* = 0.01, *t* = −13.38, *p* < 0.001, η^2^ = 0.05), indicating that psychological flexibility moderated the association between academic worry and psychological distress. The inclusion of the interaction term contributed an additional 5.0% of explained variance (Δ*R*^2^ = 0.05, 95% CI [0.05, 0.06]).

A simple slope analysis further revealed that academic worry was positively associated with psychological distress at both low and high levels of psychological flexibility, but the association was substantially attenuated at higher flexibility. Specifically, when psychological flexibility was low, academic worry was strongly associated with distress (−1 *SD*; β = 0.06, *SE* = 0.02, 95% CI [0.056, 0.64], *t* =28.86. *p* < 0.001); when psychological flexibility was high, the effect remained significant but was weaker (+1 *SD*; β = 0.25, *SE* = 0.02, 95% CI [0.22, 0.29], *t* =13.23. *p* < 0.001).

A Johnson–Neyman analysis (A. F. Hayes, 2022) indicated that the conditional effect of academic worry on distress was significant for most observed levels of psychological flexibility and became non-significant only within a narrow high-flexibility range [2.13, 2.92] in standardized units; outside this interval, the slope of academic worry was significant at *p* < 0.05.

**Figure 1:**
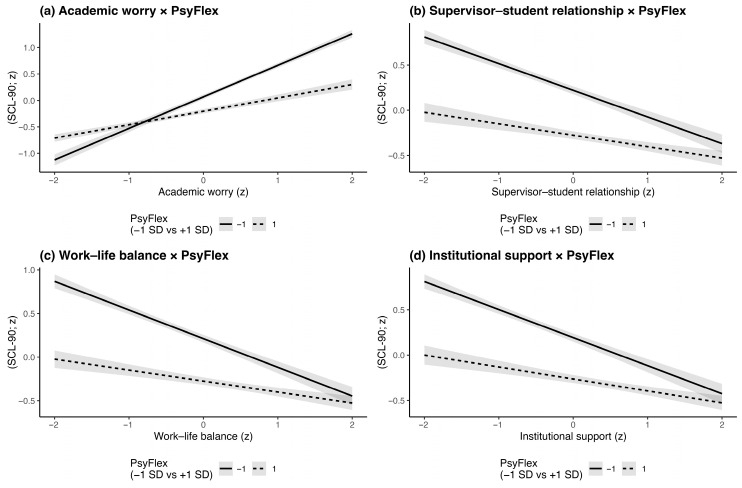
Simple slopes of postgraduate life stress variables in psychological distress (SCL-90) by psychological flexibility. The plots depict the relationship between psychological distress and the following specific stressors at high (+1 *SD*) and low (−1 *SD*) levels of psychological flexibility: (**a**) Academic worry, (**b**) Supervisor–student relationship, (**c**) Work–life balance, and (**d**) Institutional support. PsyFlex reflected psychological flexibility.

In summary, psychological flexibility significantly moderated the association between academic worry and psychological distress: academic worry was positively associated with distress at both low and high levels of psychological flexibility, but the association was substantially weaker at higher flexibility (Figure 1a).

#### 3.2.2. Psychological Flexibility as a Moderator of the Relationship Between Supervisor–Student Relationship and Psychological Health

The regression analysis examining the moderating effect of psychological flexibility on the association between supervisor–student relationship and psychological health was statistically significant, *R*^2^ = 0.153, *F* (3, 3749) = 226.07, *p* < 0.001, indicating that approximately 15.3% of the variance in the psychological distress was explained by the model. The supervisor–student relationship was significantly negatively associated with psychological distress (β = −0.21, *SE* = 0.02, *t* = −13.25, *p* < 0.001, η^2^ = 0.05), suggesting that students who perceived higher-quality supervisor–student relationships reported lower levels of psychological symptoms. Psychological flexibility was also significantly negatively associated with psychological distress (β = −0.25, *SE* = 0.02, *t* = −15.62, *p* < 0.001, η^2^ = 0.11). Among covariates, master degree stage (β = −0.04, *SE* = 0.01, *t* = −3.39, *p* < 0.001) was significantly associated with psychological distress.

The interaction term between supervisor–student relationship and psychological flexibility was significant (β = 0.08, *SE* = 0.01, *t* = 6.34, *p* < 0.001, η^2^ = 0.01), indicating that psychological flexibility positively moderated the relationship between supervisor–student relationship and psychological distress. The inclusion of the interaction term increased the explained variance by 1.0% (Δ*R*^2^ = 0.01, 95% CI [0.01, 0.02]).

Simple slope analysis further revealed that at low levels of psychological flexibility (−1 *SD*), the negative association between supervisor–student relationship and psychological distress was significant (β = −0.33, *p* < 0.001). At high levels of psychological flexibility (+1 *SD*), this negative association became weaker but remained statistically significant (β = −0.16, *p* < 0.001). As shown in Figure 1b, individuals with lower psychological flexibility benefited more from supportive supervisor–student relationships in maintaining psychological health, whereas those with higher psychological flexibility exhibited greater internal emotional regulation and were less dependent on external relational support. A Johnson–Neyman analysis further showed that the conditional association between the supervisor–student relationship and psychological distress was statistically significant for most observed values of psychological flexibility and became non-significant only within a high-flexibility interval [1.82, 3.73] in standardized units.

Psychological flexibility moderated the association between the supervisor–student relationship and psychological distress: the negative association was stronger at lower levels of psychological flexibility and weaker at higher levels of psychological flexibility (Figure 1b).

#### 3.2.3. Psychological Flexibility as a Moderator of the Relationship Between Work–Life Balance and Psychological Health

The regression model testing the moderating effect of psychological flexibility on the relationship between work–life balance and psychological distress was statistically significant (*R*^2^ = 0.16, *F* (3, 3749) = 241.19, *p* < 0.001, indicating that the model explained 16.2% of the variance in the SCL-90 total score. Work–life balance was significantly negatively associated with psychological distress (β = −0.23, *SE* = 0.02, *t* = −14.43, *p* < 0.001, η^2^ = 0.05), and psychological flexibility was also significantly negatively associated with psychological distress (β = −0.24, *SE* = 0.02, *t* = −15.42, *p* < 0.001, η^2^ = 0.11). No covariates were significantly associated with psychological distress.

Importantly, the interaction between work–life balance and psychological flexibility was significant (β = 0.10, *SE* = 0.01, *t* = 7.65, *p* < 0.001, η^2^ = 0.02), indicating a significant moderating effect. The inclusion of this interaction term increased the explained variance by 2.0% (Δ*R*^2^ = 0.02, 95% CI [0.01, 0.02]).

Simple slope analysis revealed that the association between work–life balance and psychological distress varied across levels of psychological flexibility. Specifically, when psychological flexibility was low (−1 *SD*), higher work–life balance was more strongly associated with lower psychological distress (β = −0.34, *p* < 0.001). When psychological flexibility was high (+1 *SD*), this negative association remained statistically significant but was attenuated (β = −0.14, *p* < 0.001), as illustrated in Figure 1c. A Johnson–Neyman analysis further identified the range of psychological flexibility in which the conditional association between work–life balance and psychological distress was statistically significant for most observed values of psychological flexibility and became non-significant only within a high-flexibility interval [1.71, 3.08] in standardized units.

In summary, psychological flexibility significantly moderated the association between work–life balance and psychological health, such that the negative association between work–life balance and distress was stronger at lower levels of psychological flexibility and weaker at higher levels.

#### 3.2.4. Psychological Flexibility as a Moderator of the Relationship Between Institutional Supports and Psychological Health

The regression model testing the moderating effect of psychological flexibility on the association between institutional supports and psychological distress was statistically significant, *R*^2^ = 0.15, *F* (3, 3749) = 225.72, *p* < 0.001, indicating that the model explained 15.3% of the variance in the SCL-90 total score. Institutional supports were significantly negatively associated with psychological distress (β = −0.22, *SE* = 0.02, *t* = −13.48, *p* < 0.001, η^2^ = 0.05), suggesting that stronger perceived institutional supports was associated with lower levels of psychological distress. Psychological flexibility also showed a significantly negative association with psychological distress (β = −0.23, *SE* = 0.02, *t* = −13.91, *p* < 0.001, η^2^ = 0.11). Among covariates, master degree stage (β = −0.03, SE = 0.01, t = −2.64, *p* < 0.001) was significantly associated with psychological distress.

Importantly, the interaction between institutional supports and psychological flexibility was significant (β = 0.09, *SE* = 0.01, *t* = 6.62, *p* < 0.001, η^2^ = 0.01), indicating that psychological flexibility moderated the relationship between institutional supports and psychological distress. The inclusion of this interaction term increased the explained variance by 1.0% (Δ*R*^2^ = 0.01, 95% CI [0.01, 0.02]).

Simple slope analysis further revealed that the association between institutional supports and psychological distress varied across levels of psychological flexibility. Specifically, As shown in Figure 1d, when psychological flexibility was low(−1 *SD*), perceived institutional supports had the strongest negative association with psychological distress (β = −0.32, *p* < 0.001), when psychological flexibility was high (+1 *SD*), this effect weakened but remained statistically significant (β = −0.14, *p* < 0.001). A Johnson–Neyman analysis further identified the range of psychological flexibility in which the conditional association between work–life balance and psychological distress was statistically significant for most observed values of psychological flexibility and became non-significant only within a high-flexibility interval [1.84, 3.60] in standardized units.

In summary, psychological flexibility significantly moderated the association between perceived institutional supports and psychological distress, such that higher institutional supports were more strongly associated with lower psychological distress at lower levels of psychological flexibility, while the association was weaker at higher levels of psychological flexibility.

## 4. Discussion

The present study found that the four dimensions of life stress (i.e., academic worry, supervisor–student relationship, work–life balance, and institutional support) were all significantly associated with the psychological distress of medical postgraduates. Specifically, higher levels of academic worry were related to more severe psychological symptoms, whereas better supervisor–student relationships, greater work–life balance, and stronger institutional support were associated with better mental health, thus supporting Hypothesis 1. Psychological flexibility was negatively associated with psychological distress, supporting Hypothesis 2. Furthermore, psychological flexibility significantly moderated all four associations between life stressors and psychological distress, providing support for Hypothesis 3.

Among the four stressor–psychological distress associations, the moderation by psychological flexibility was most pronounced for academic worry (η^2^ = 0.16). This pattern suggests that the strength of the worry–distress association varies more across levels of psychological flexibility than do the associations involving more externally anchored stressors (e.g., relational or institutional conditions). Within the ACT framework, this finding is consistent with the view that psychological flexibility is centrally relevant to how individuals relate to internally generated cognitive experiences (such as worry), through processes like cognitive diffusion, acceptance, and values-consistent action, which may be especially pertinent when stress is predominantly cognition-based (Feather & Williams, 2022; Hsu et al., 2023). Recent work has similarly highlighted psychological flexibility as a key process linked to distress across contexts and as a modifiable target in ACT-informed interventions (Westhoff et al., 2024; Zhang et al., 2024).

Among these effects, the moderating role of psychological flexibility was strongest in the pathway involving academic worry, indicating that psychological flexibility may be particularly effective in regulating internal, cognition-related stress. This finding extends prior research that has primarily focused on psychological flexibility as a moderator of external stressors, such as environmental pressure or lack of social support. The results suggest that psychological flexibility may also play a key role in coping with endogenous cognitive stress, highlighting an important direction for future research into its moderating effects and boundary conditions.

### 4.1. Theoretical Contributions

This study makes several theoretical contributions. First, previous research has mainly examined the moderating effects of psychological flexibility in clinical populations, with limited attention to its function in non-clinical but high-stress groups (Stockton et al., 2019). By incorporating psychological flexibility into the life stress–mental health model of medical postgraduates, this study provides empirical evidence for its universality and adaptability in a non-clinical, high-risk population. This extends the theoretical application of psychological flexibility across different contexts.

Second, this study refines the conceptualization of postgraduate life stress by identifying four distinct situational dimensions and demonstrates the heterogeneity of their relationships with mental health. By further testing the differentiated moderating effects of psychological flexibility across these stress domains, the findings expand the applicability of the stress–resource interaction model within the graduate student population. In addition, we found an interesting intersection for the effect of academic worries on distress under higher and lower levels of psychological flexibility (Denckla et al., 2018). This could be related to affective instability and behavioral instability when individuals’ self-perceived academic stress is lower than average. However, further control of behavioral confounding factors and longitudinal follow-ups should be involved to examine this proposed theory.

Third, the results indicate that psychological flexibility may exert stronger negative moderating effects on internal cognitive stressors, such as academic worry, compared to external contextual stressors. This provides new insight into the role of psychological flexibility in facilitating adaptive responses to internalized stress, self-evaluation, and anticipatory anxiety, thereby deepening the theoretical understanding of its potential protective and regulatory functions.

### 4.2. Practical Implications

This study provides several meaningful implications for graduate education and administrative practice in higher education.

Drawing on the four-dimensional stress model, universities could develop targeted scales to evaluate perceived stress of graduate students, addressing the limitations that traditional assessments focus mainly on symptoms and lack specificity regarding stressors (Dyrbye & Shanafelt, 2011). Such an instrument would support more precise early-warning systems and enable student health administrators to perform evidence-based and individualized interventions.

A further implication concerns the refinement of supervisory systems. Although graduate students generally report positive experiences with their advisors, gaps remain in areas such as communication frequency, emotional support, and career guidance when compared with international standards. Universities may consider developing formative evaluation and training for supervisors, with the aim of strengthening their overall supervising competencies.

Another important direction involves fostering work–life balance through flexible training pathways, including optimized coordination of clinical duties and research tasks for medical postgraduates. Establishing integrated health-support platforms and offering diverse well-being programs, such as art-based activities, physical exercise, and mindfulness, can embed mental health cultivation within academic development and promote more holistic student growth.

Finally, there is a need to enhance both academic and mental health support systems. Providing resources for academic exchange and research funding, alongside high-quality mental health education and professional counseling services, can create a more supportive environment. Building clear help-seeking channels and cross-departmental support networks will further strengthen institutional capacity to safeguard graduate students’ well-being.

Notably, psychological flexibility emerged as a particularly powerful protective factor against psychological distress (Marshall & Brockman, 2016). Reducing experiential avoidance and cognitive fusion encourages more adaptive coping. Integrating psychological flexibility into graduate training may therefore be highly beneficial. A dual-track approach could be adopted by embedding practical techniques (e.g., cognitive defusion strategies for handling rejection-related anxiety) into research skills curricula, while offering scenario-based workshops through campus mental health services to enhance emotional acceptance and problem-solving abilities.

### 4.3. Limitations and Future Directions

Although the present study revealed the important moderating role of psychological flexibility in the relationship between life stress and mental health among medical postgraduates, several limitations should be noted.

First, the study relied on a cross-sectional, self-report design, which limits inferences about the temporal ordering of variables and may be influenced by response biases (e.g., social desirability). Future research could use longitudinal or intervention designs and incorporate multi-method assessments (e.g., behavioral indicators or physiological measures) to strengthen the robustness of the findings.

Second, the current study utilized single population sample for both exploratory and confirmatory factor analysis due to sample restrictions. We encourage future studies to use external sample to validate the Postgraduate Stress and Support Questionnaire to improve robustness.

Finally, future research could integrate psychological flexibility with other positive psychological resources, such as resilience and emotion regulation ability, within a unified framework to construct a more comprehensive theoretical model for understanding and promoting mental health among postgraduate students.

## Figures and Tables

**Table 1:** Relationships among postgraduate life variables, psychological distress, and psychological flexibility (*n* = 3753).

Measure	*M*	*SD*	1	2	3	4	5	6
1 Psychological flexibility	5.0	0.6	-	-	-	-	-	-
2 Academic worry	2.8	0.9	−0.40 ***	-	-	-	-	-
3 Supervisor-student relationship	4.0	0.6	−0.33 ***	−0.55 ***	-	-	-	-
4 Work–life balance	3.5	0.7	/0.32 ***	−0.56 ***	/0.60 ***	-	-	-
5 Institutional supports	3.8	0.7	/0.40 ***	−0.54 ***	/0.55 ***	/0.58 ***	-	-
6 Psychological distress	1.2	0.3	−0.32 ***	/0.47 ***	−0.30 ***	−0.30 ***	−0.31	-

Note. *** *p* < 0.001.

## Data Availability

The original data presented in the study are openly available in OSF at https://osf.io/mhgnc/overview?view_only=3df9a0cdb6524cfeb85e90dac28d0680 (accessed on 19 November 2025).

## Supplementary Materials

The following supporting information can be downloaded at: https://www.mdpi.com/article/10.3390/bs16030374/s1.

## Abbreviations

The following abbreviations are used in this manuscript:

ACT	Acceptance and Commitment Therapy
SCL-90	Symptom Checklist-90
Comp ACT	Comprehensive Assessment of ACT progress
EFA	Exploratory Factor Analysis
KMO	The Kaiser–Meyer–Olkin Measure
CFA	Confirmatory Factor Analysis

## Appendix A

**Table A1:** Exploratory factor analysis.

Item	Factor 1	Factor 2	Factor 3	Factor 4	Communality
1. I have concerns about future job opportunities	0.87				0.72
2. I have anxiety over personal competence and achievements	0.84				0.66
3. I have financial security concerns	0.83				0.62
4. I have worries about devaluation of graduate degrees	0.79				0.63
5. I have fear of delayed graduation	0.75				0.55
6. I have mental health apprehensions	0.57				0.49
7. I have concerns about negative work–life integration	0.54				0.60
8. I have anxiety regarding delayed stipend payments	0.52				0.33
9. I have a good advisor-advisee relationship		0.97			0.83
10. I can feel my supervisor’s support		0.96			0.81
11. I can feel recognition from supervisor		0.87			0.73
12. I have good research group welfare		0.71			0.64
13. I can feel helpful instruction in my research group		0.44			0.55
14. I have conference participation opportunities		0.42			0.49
15. I have work autonomy			0.94		0.74
16. I have workload manageability			0.93		0.74
17. I have leave flexibility			0.90		0.74
18. I have good work–life balance			0.77		0.75
19. I have good social environment			0.51		0.50
20. My university provides individual psychological counseling				0.98	0.84
21. My university provides mental health activities				0.95	0.85
22. My university provides mental health services				0.85	0.72
23. My university supports student work–life balance				0.74	0.76
Proportion of variance explained	42.90	9.24	7.42	6.16	
Cumulative proportion of variance explained	42.90	52.14	59.55	65.71	

Note. Factor 1 “Academic Worry” comprises items 1–8; Factor 2 “ Supervisor–student Relationship” includes items 9–14; Factor 3 “Work–Life Balance” contains items 15–19; Factor 4 “Institutional Support” encompasses items 20–23.
