# Supplementary material for: Psychological Flexibility Moderates the Association Between Multidimensional Stress and Psychological Distress in Medical Postgraduates: A Multi-Center Cross-Sectional Study
Source: Behav Sci (Basel). 2026 Mar 6;16(3):374. doi: 10.3390/bs16030374 (PMC13023548; doi:10.3390/bs16030374)
Supplement: Supplementary file 1 [file behavsci-16-00374-s001.zip › behavsci-4097804-supplementary.pdf]

1 The relationship between life-related stress on anxiety of medical postgraduates: moderation role of psychological flexibility

Four separate moderation models were estimated to examine whether psychological flexibility moderated the associations between each stress-related variable and anxiety.

(a) Academic worry × Psychological flexibility

In the academic worry model, academic worry was positively associated with anxiety (β = 0.77, SE = 0.05, $p < .001$, 95% CI [0.67, 0.87]), and psychological flexibility was also positively associated with anxiety (β = 0.27, SE = 0.03, $p < .001$, 95% CI [0.21, 0.33]). The academic worry × psychological flexibility interaction was statistically significant (β = −0.12, SE = 0.01, $p < .001$, 95% CI [−0.14, −0.10]).

Simple slope analyses indicated that academic worry was positively associated with anxiety at both low (−1 SD) and high (+1 SD) levels of psychological flexibility. Specifically, at low psychological flexibility, higher academic worry was associated with higher anxiety (β = 0.24, SE = 0.01, 95% CI [0.22, 0.25], $p < .001$). At high psychological flexibility, this positive association remained statistically significant but was reduced in magnitude (β = 0.10, SE = 0.01, 95% CI [0.08, 0.11], $p < .001$).

(b) Supervisor–student relationship × Psychological flexibility

In the supervisor–student relationship model, the supervisor–student relationship was negatively associated with anxiety (β = −0.53, SE = 0.07, $p < .001$, 95% CI [−0.68, −0.38]), and psychological flexibility was negatively associated with anxiety (β = −0.47, SE = 0.06, $p < .001$, 95% CI [−0.58, −0.35]). The supervisor–student relationship × psychological flexibility interaction was statistically significant (β = 0.08, SE = 0.01, $p < .001$, 95% CI [0.05, 0.11]).

Simple slope analyses showed that the supervisor–student relationship was negatively associated with anxiety at both levels of psychological flexibility. At low psychological flexibility (−1 SD), a more supportive supervisor–student relationship was associated with lower anxiety (β = −0.17, SE = 0.01, 95% CI [−0.19, −0.14], $p < .001$). At high psychological flexibility (+1 SD), this negative association remained statistically significant but was weaker (β = −0.07, SE = 0.01, 95% CI [−0.10, −0.04], $p < .001$).

(c) Work–life balance × Psychological flexibility

In the work–life balance model, work–life balance was negatively associated with anxiety (β = −0.53, SE = 0.06, p < .001, 95% CI [−0.65, −0.41]), and psychological flexibility was negatively associated with anxiety (β = −0.43, SE = 0.04, $p < .001$, 95% CI [−0.51, −0.34]). The work–life balance × psychological flexibility interaction was statistically significant (β =

0.09, SE = 0.01, $p < .001$, 95% CI [0.06, 0.11]).

Simple slope analyses indicated that work–life balance was negatively associated with anxiety at both low and high levels of psychological flexibility. At low psychological flexibility (−1 SD), better work–life balance was associated with lower anxiety (β = −0.15, SE = 0.01, 95% CI [−0.18, −0.13], $p < .001$). At high psychological flexibility (+1 SD), this negative association remained statistically significant but was smaller in magnitude (β = −0.06, SE = 0.01, 95% CI [−0.08, −0.03], $p < .001$).

(d) Institutional support × Psychological flexibility

In the institutional support model, institutional support was negatively associated with anxiety (β = −0.43, SE = 0.06, $p < .001$, 95% CI [−0.54, −0.31]), and psychological flexibility was negatively associated with anxiety (β = −0.37, SE = 0.05, $p < .001$, 95% CI [−0.46, −0.28]). The institutional support × psychological flexibility interaction was statistically significant (β = 0.07, SE = 0.01, $p < .001$, 95% CI [0.04, 0.09]).

Simple slope analyses showed that institutional support was negatively associated with anxiety at both levels of psychological flexibility. At low psychological flexibility (−1 SD), greater institutional support was associated with lower anxiety (β = −0.14, SE = 0.01, 95% CI [−0.16, −0.11], $p < .001$). At high psychological flexibility (+1 SD), this negative association remained statistically significant but weaker (β = −0.06, SE = 0.01, 95% CI [−0.08, −0.04], $p < .001$).

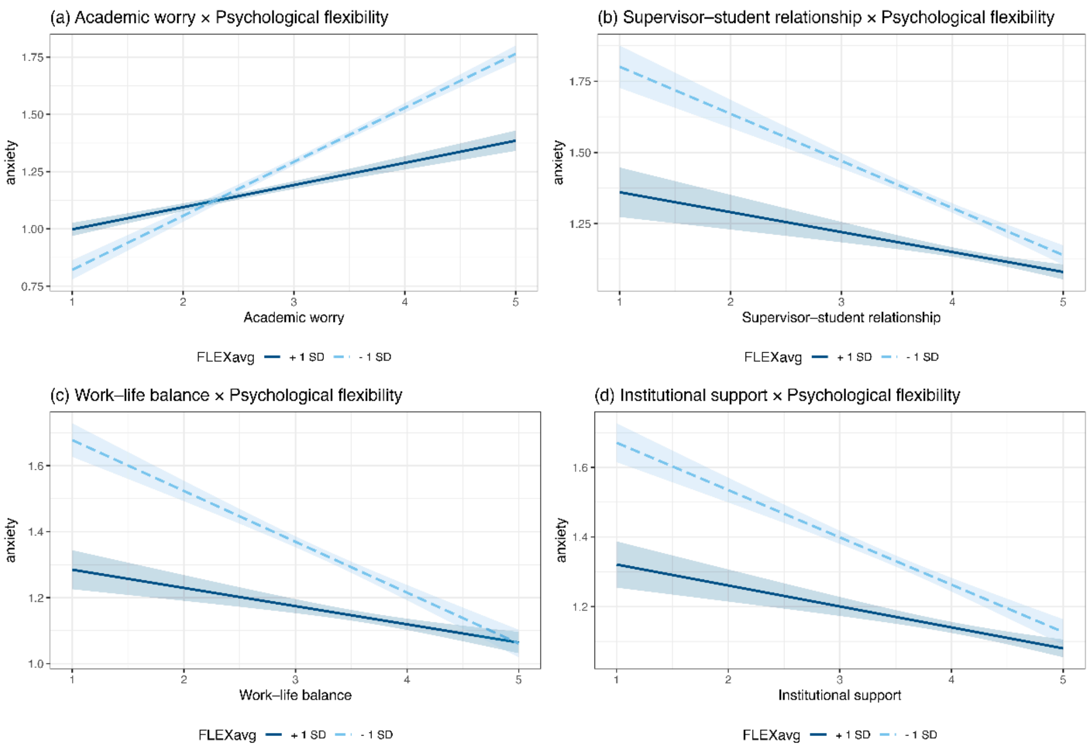

Figure S1. Interaction effects of psychological flexibility on the associations between four stress-related factors and psychological anxiety. Panels show the conditional associations between (a) academic worry, (b) supervisor–student relationship, (c) work–life balance, and (d) institutional support and anxiety at low (−1 SD) and high (+1 SD) levels of psychological flexibility. Shaded areas represent 95% confidence intervals.

2 The relationship between life-related stress on depression of medical postgraduates: moderation role of psychological flexibility

Four separate moderation models were estimated to examine whether psychological flexibility moderated the associations between each stress-related variable and depression.

(a) Academic worry × Psychological flexibility

In the academic worry model, academic worry was positively associated with depression (β = 1.09, SE = 0.06, $p < .001$, 95% CI [0.98, 1.20]), and psychological flexibility was positively associated with depression (β = 0.37, SE = 0.04, $p < .001$, 95% CI [0.30, 0.44]). The academic worry × psychological flexibility interaction was statistically significant (β = −0.18, SE = 0.01, $p < .001$, 95% CI [−0.20, −0.15]).

Simple slope tests indicated that academic worry was positively associated with depression at both low (−1 SD) and high (+1 SD) levels of psychological flexibility. Specifically, at low psychological flexibility, higher academic worry was associated with higher depression (β = 0.32, SE = 0.01, 95% CI [0.30, 0.34], $p < .001$). At high psychological flexibility, this positive

association remained statistically significant but was smaller in magnitude (β = 0.12, SE = 0.01, 95% CI [0.10, 0.14], $p < .001$).

(b) Supervisor–student relationship × Psychological flexibility

In the supervisor–student relationship model, the supervisor–student relationship was negatively associated with depression (β = −0.80, SE = 0.09, $p < .001$, 95% CI [−0.97, −0.63]), and psychological flexibility was negatively associated with depression (β = −0.73, SE = 0.07, $p < .001$, 95% CI [−0.87, −0.59]). The supervisor–student relationship × psychological *flexibility* interaction was statistically significant (β = 0.13, SE = 0.02, $p < .001$, 95% CI [0.09, 0.16]).

Simple slope tests showed that the supervisor–student relationship was negatively associated with depression at both levels of psychological flexibility. At low psychological flexibility (−1 SD), a more supportive supervisor–student relationship was associated with lower depression (β = −0.23, SE = 0.02, 95% CI [−0.26, −0.20], $p < .001$). At high psychological flexibility (+1 SD), this negative association remained statistically significant but was smaller (β = −0.08, SE = 0.02, 95% CI [−0.12, −0.05], $p < .001$).

(c) Work–life balance × Psychological flexibility

In the work–life balance model, work–life balance was negatively associated with depression (β = −0.79, SE = 0.07, $p < .001$, 95% CI [−0.93, −0.65]), and psychological flexibility was negatively associated with depression (β = −0.65, SE = 0.05, $p < .001$, 95% CI [−0.75, −0.55]). The work–life balance × psychological flexibility interaction was statistically significant (β = 0.13, SE = 0.01, $p < .001$, 95% CI [0.10, 0.16]).

Simple slope tests indicated that work–life balance was negatively associated with depression at both low and high levels of psychological flexibility. At low psychological flexibility (−1 SD), better work–life balance was associated with lower depression (β = −0.22, SE = 0.01, 95% CI [−0.25, −0.20], $p < .001$). At high psychological flexibility (+1 SD), this negative association remained statistically significant but was weaker (β = −0.08, SE = 0.01, 95% CI [−0.10, −0.05], $p < .001$).

(d) Institutional support × Psychological flexibility

In the institutional support model, institutional support was negatively associated with depression (β = −0.75, SE = 0.07, $p < .001$, 95% CI [−0.88, −0.61]), and psychological flexibility was negatively associated with *depression* (β = −0.65, SE = 0.05, $p < .001$, 95% CI [−0.76, −0.55]). The institutional support × psychological flexibility interaction was statistically significant (β = 0.12, SE = 0.01, $p < .001$, 95% CI [0.09, 0.15]).

Simple slope tests showed that institutional support was negatively associated with depression

at both levels of psychological flexibility. At low psychological flexibility (−1 SD), greater institutional support was associated with lower depression (β = −0.21, SE = 0.01, 95% CI [−0.23, −0.18], $p < .001$). At high psychological flexibility (+1 SD), this negative association remained statistically significant but was weaker (β = −0.07, SE = 0.01, 95% CI [−0.09, −0.04], $p < .001$).

Overall, the conditional association patterns for the SCL-90 total score were largely consistent with those observed for the anxiety and depression subscales. Specifically, for academic worry, the SCL-90 total score model showed significant positive simple slopes at both low and high levels of psychological flexibility, with a reduced slope magnitude at higher flexibility. This mirrors the anxiety and depression models, in which academic worry was also positively associated with each symptom domain at both levels of psychological flexibility, again with smaller slopes at higher flexibility. Accordingly, the updated results suggest that the moderation pattern observed for the global distress indicator is largely consistent with symptom-specific outcomes, with differences primarily reflected in the magnitude (rather than the direction or presence) of the conditional associations across outcomes.

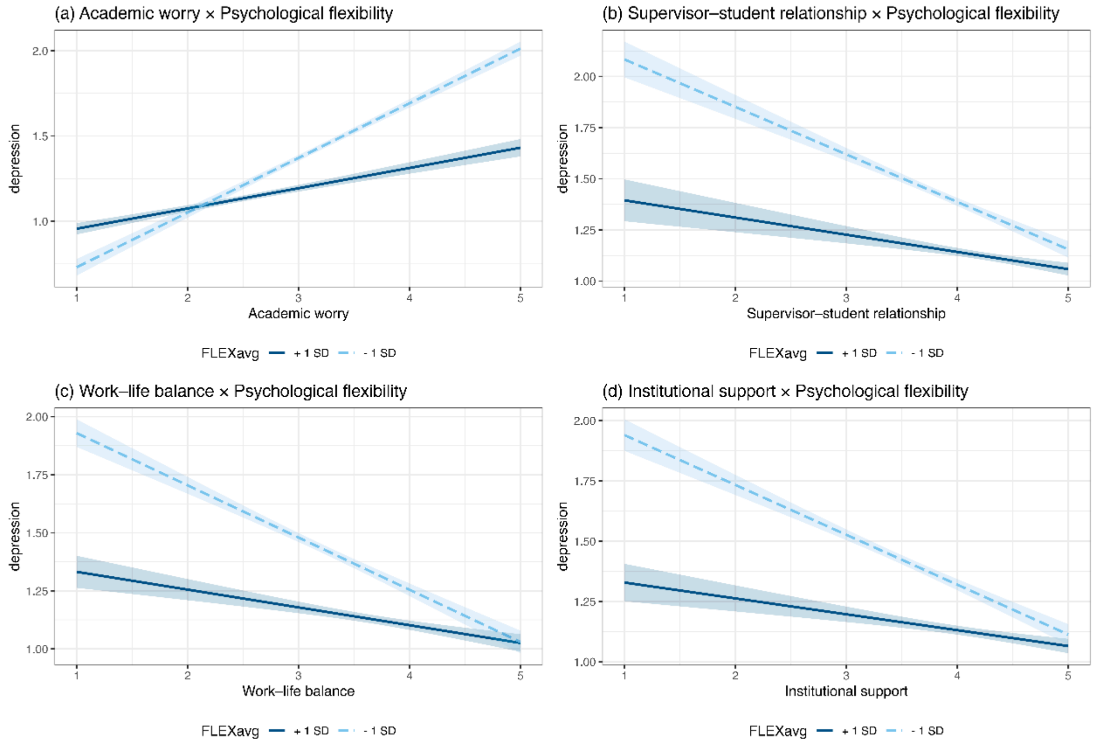

Figure S2. Interaction effects of psychological flexibility on the associations between four stress-related factors and psychological distress. Panels show the conditional associations between (a) academic worry, (b) supervisor–student relationship, (c) work–life balance, and (d) institutional support and anxiety at low (−1 SD) and high (+1 SD) levels of psychological flexibility. Shaded areas represent 95% confidence intervals.
